# Cytoplasmic Viruses: Rage against the (Cellular RNA Decay) Machine

**DOI:** 10.1371/journal.ppat.1003762

**Published:** 2013-12-05

**Authors:** Stephanie L. Moon, Jeffrey Wilusz

**Affiliations:** Department of Microbiology, Immunology and Pathology, Colorado State University, Fort Collins, Colorado, United States of America; Columbia University, United States of America

As our appreciation increases for the pervasive nature of transcription in the cell, so too has our appreciation for the major role of RNA decay/stability in regulating both the quantity and the quality of gene expression. As soon as viral RNAs appear in the cell, they must be prepared to combat or avoid cellular RNA decay pathways. This review describes the myriad ways that viruses deal with the general host RNA decay machinery that is active in the cell immediately upon viral infection—turning what, at first, appears to be very hostile territory for a foreign transcript into a sort of “promised land” for viral gene expression. It is interesting to note that cells likely try to adapt to this viral interference with the general RNA decay machinery by inducing a variety of novel RNases as part of a molecular arms race.

## The Host RNA Decay Machinery Is a Major Impediment to Cytoplasmic Viruses

The cellular RNA decay machinery constantly monitors transcripts, from the time they are synthesized in the nucleus until the end of their lifespan in the cytoplasm. Aberrant products of transcription initiation (e.g. PROMPTS), capping, and termination are quickly degraded by nuclear RNA quality control surveillance complexes. Misfolded, “mis”-translated (e.g. mRNAs with a premature termination codon), and mispackaged mRNAs are also quickly degraded in the cytoplasm. In addition to removing aberrant mRNAs, up to 50% of cellular gene expression may be controlled by changes in mRNA stability. When a typical cellular mRNA is targeted for decay, it initially undergoes deadenylation—the removal of the 3′ poly(A) tail. The mRNA is then subject to processive exonucleolytic degradation in either the 3′-5′ direction by the exosome or Dis3L2, or it is marked by the LSm1-7/Pat1 complex for decapping by Dcp1/2 and degraded in the 5′-3′ direction by Xrn1 [1].

When the transcripts of cytoplasmic viruses are generated, they must actively avoid or overcome the assault by these aggressive cellular mRNA decay complexes in order to be translated and effectively generate virions. It should be easy for the cellular RNA decay machinery to recognize these foreign transcripts—typical host mRNAs, for example, are assembled into characteristic ribonucleoprotein complexes in the nucleus, but the RNAs of cytoplasmic viruses never have this opportunity. In addition, some viral RNAs do not have 5′ caps or poly(A) tails, and some have multiple open reading frames or long 3′ UTRs, which should target them for nonsense-mediated decay. Yet, the transcripts generated by cytoplasmic viruses survive and flourish in this hostile cytoplasmic environment. Interestingly, viruses do more than simply mimic host mechanisms like polyadenylation, triple helix structures, and 5′ capping to protect their transcripts from host exonucleases [2]. Cytoplasmic viruses use diverse strategies to overcome the odds and trick the host into ignoring or even, preferentially, stabilizing their transcripts. Such viral RNA trickery is a fascinating aspect of host–virus interaction that we are just now beginning to understand.

## Viral RNAs Versus Cellular RNA Decay Factors: Destruction and Deception

Several cytoplasmic viruses directly repress key aspects of the cellular RNA decay machinery to promote viral RNA stability. Picornaviruses use an aggressive mechanism for suppression of host RNA decay factors. Xrn1, Dcp1, Dcp2, Pan3 (a deadenylase), and AUF1 (a factor that targets RNAs for decay) are rapidly degraded during poliovirus or human rhinovirus infections by viral proteases and/or the host cell proteasome [3], [4]. The importance of this suppression has recently been demonstrated through the negative effects that AUF1 has on picornavirus replication [5]. The dispersal of P-bodies (cytoplasmic aggregates of host RNA decay factors) in several viral infections is also evidence of disruption of cellular RNA decay activities [6]. Alternatively, arthropod-borne flaviviruses, including West Nile virus (WNV), generate a large amount of a short subgenomic RNA (sfRNA) by stalling the Xrn1 5′-3′ exoribonuclease on pseudoknot-like structures in the viral 3′ UTR [7], [8]. Interestingly, stalling of Xrn1 on the viral 3′ UTR also inactivates the enzyme, presumably due to its slow release from sfRNA [9]. The repression of Xrn1 by the generation of sfRNA is very important in a flavivirus infection. WNV variants that cannot effectively form sfRNA show defects in viral growth in certain cell types and reduced cytopathology [8], [10]. Disparate RNA viruses have, therefore, evolved unique mechanisms by which they disarm host RNA decay pathways by inactivating or proteolytically degrading important nucleases to promote productive viral infections.

Paradoxically, several cytoplasmic viruses even turn a host RNA decay factor into a stabilizing factor. Several viruses have developed a way to steal the host LSm1-7 complex that normally marks deadenylated transcripts for 5′-3′ degradation. Brome Mosaic virus genomic RNA has internal poly(A) tracts and tRNA-like structures in the 3′ UTR that facilitate LSm1-7 binding to promote viral translation and replication [11]. Hepatitis C virus (HCV) RNAs have similarly been shown to bind LSm1-7, and knockdown of the RNA decay factors LSm1 and PatL1 dramatically reduces HCV translation and replication [12]. Finally, many transcripts of the cytoplasmic DNA orthopoxviruses have unique, nontemplated poly(A) tracts at their 5′ ends that bind LSm1-7 and can stabilize RNAs [13]. Therefore, by attracting the LSm1-7 complex and associated factors in an unconventional fashion, viral RNAs certainly have figured out a way to make the best of what would normally be a bad situation for a transcript.

## Viral RNAs That Steal to Survive: “Borrowing” of Host RNA Stability Factors

It has been known for some time that members of the *Arenaviridae*, *Bunyaviridae*, and the nuclear *Orthomyxoviridae* families steal the 5′ capped ends of host mRNAs to incorporate this *cis*-acting stability element into their own transcripts [14]. Emerging evidence indicates that the 2′-O-methylation of cap structures is read by innate immune interferon stimulated genes (ISGs) as a way to differentiate host versus virus transcripts. Cap-stealing mechanisms used by segmented RNA viruses to generate their mRNAs circumvent this innate detection system. Furthermore, recent evidence indicates that cellular *trans*-acting factors that stabilize host transcripts are also purloined by thieving viral RNAs.

The cellular HuR protein is a well-characterized shuttling factor that promotes the stability of mRNAs by interacting with U-rich elements. Alphaviruses contain highly conserved U-rich elements or other high-affinity HuR binding sites in the 3′ UTR of their RNAs that bind HuR during infection to promote viral RNA stability and efficient virus production [15], [16]. HuR is not the only regulatory mRNA decay factor that is commandeered by cytoplasmic RNA viruses. Rabies virus glycoprotein mRNA and poliovirus transcripts steal host poly(C) binding protein 2 (PCBP2), leading to increased transcript abundance and stability [17], [18]. Usurping PCBP2 may help rabies virus tightly regulate expression of its glycoprotein to avoid host immune detection as it replicates and migrates to the central nervous system during infection. Viral RNAs may also “sponge” miRNAs (e.g. [19]) and, perhaps, cellular RNA binding proteins by sequestering these cellular factors on high affinity binding sites present on viral transcripts to promote viral-specific gene expression.

## Viral-Encoded Ribonucleases: If You Don't Like the Sandbox That You Are Playing in, Make a New One

Virally encoded endonucleases are important for many aspects of viral replication, including the fine-tuning of viral gene expression by rapidly depleting old viral mRNAs to enhance the expression of newly transcribed mRNAs [20]. In addition, to make a cell more amenable to virus production, these virally encoded nucleases may also create a new “sandbox” in the cytoplasm for viral RNAs by initiating the large-scale decay of cellular mRNAs and dramatically altering the landscape of host gene expression. Interestingly, the internal cleavage of host mRNAs by disparate betacoronaviruses, influenza viruses, vaccinia viruses, and the nuclear herpesviruses may force host exoribonucleases like Xrn1 and the exosome to divert their attention to degrading this large number of products of viral endonucleolytic decay [21]. The host RNA decay machinery may, therefore, become saturated as endonucleolytic decay products rapidly accumulate during viral infection, limiting its normal functions. Thus, virus-derived nucleases may disrupt normal gene expression and RNA decay-related quality control mechanisms to help viral RNAs escape detection by the cellular RNA decay machinery.

## You Must Decay or You Will Pay: Cytopathology Can Result from Dysregulated RNA Decay

**Figure 1:**
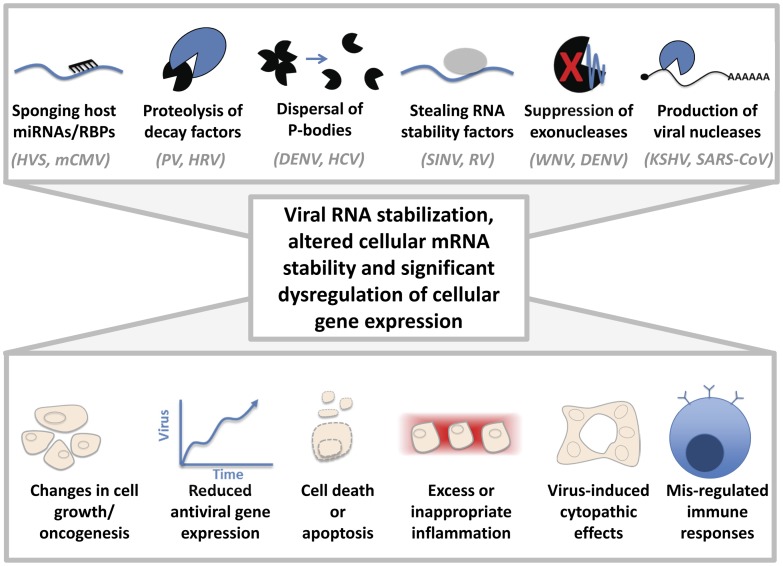
Cytoplasmic viruses may induce pathology by altering host mRNA decay pathways. Sponging of host miRNAs or RNA-binding proteins (RBPs), proteolysis of cellular decay factors, dispersal of processing (P)-bodies, stealing host RNA stability factors, suppressing exonucleases, and/or the production of viral nucleases can dramatically affect the regulation of cellular gene expression. Representative examples of viruses that are associated with these mechanisms are indicated. HVS: Herpesvirus saimiri, mCMV: Murine cytomegalovirus, PV: Poliovirus, HRV: Human rhinovirus, DENV: Dengue virus, HCV: Hepatitis C virus, SINV: Sindbis virus, RV: Rabies virus, WNV: West Nile virus, KSHV: Kaposi sarcoma herpesvirus, SARS-CoV: severe acute respiratory syndrome coronavirus. Changes in host gene expression could lead to altered cell growth or oncogenesis, viral proliferation due to lack of an antiviral response, cell death/apoptosis, cytopathic effects, or excess/inappropriate inflammation, as observed in “cytokine storms” during flavivirus infections.

Considering the importance of RNA stability in regulating transcript abundance, the inactivation or commandeering of cellular RNA decay factors by viruses is likely to significantly alter host gene expression. How might changes in host mRNA stability contribute to virus-induced pathology during infection (Figure 1)? One example of this phenomenon is that wild-type Kunjin virus was significantly more pathogenic in both tissue culture and mouse models of infection than a mutant virus incapable of forming sfRNA [7]. Inactivation of Xrn1 by Kunjin virus sfRNA likely causes the stabilization and increase in abundance of numerous short-lived host transcripts, including chemokines, cytokines, and cell cycle regulators [6]. Dysregulation of these factors by Xrn1 inhibition may lead to excessive inflammation, dysregulation of the immune response, and/or changes in cell growth. Recent work in yeast has demonstrated the ability of Xrn1 to enter the nucleus and influence transcription rates, thus acting as a link between RNA decay and transcription [22]. Excitingly, the authors found that the exonucleolytic activity of Xrn1 was also required for the coupling between transcription and mRNA decay. Could sfRNA-mediated inactivation of Xrn1 cause a defect in the coordination of RNA decay and transcription in the host? If so, this could dramatically alter host gene expression and directly influence pathogenesis.

## Concluding Remarks

Viral RNAs have evolved a wide variety of mechanisms to successfully interface with the host RNA decay machinery. In fact, some of the most important questions in this field have yet to be answered. What are the consequences of viral inactivation of decay factors like Xrn1 in terms of disease? Can viruses also influence host transcription by manipulating RNA decay pathways to short-circuit feedback regulatory mechanisms? How do virus-induced changes in RNA decay pathways interface with potential changes in innate immune responses?

Virus families often use conserved strategies to evade the cellular RNA decay machinery. Therefore, perhaps researchers can develop effective, broad-spectrum antivirals to disarm these strategies and destabilize viral RNAs. Future research in this burgeoning field will likely uncover novel mechanisms of the regulation of host and viral gene expression and facilitate new methods for treating viral diseases.
